# Supplementary material for: Placental sex-dependent spermine synthesis regulates trophoblast gene expression through acetyl-coA metabolism and histone acetylation
Source: Commun Biol. 2022 Jun 15;5:586. doi: 10.1038/s42003-022-03530-6 (PMC9200719; doi:10.1038/s42003-022-03530-6)
Supplement: Supplementary file 2 — Supplementary Information [file 42003_2022_3530_MOESM2_ESM.pdf]

A

	Reads per tissue (RNA-seq) or Number of nuclei (out of a total of 96) called											
	SMS<br>SNP Position Chr X:21940733 (A/G)						TIMM17B<br>SNP Position Chr X:48894188 (G/A)					
	RNAseq		Single Nuc			Cord DNA	RNAseq		Single Nuc			Cord DNA
Sample	Reference	Alternate	Reference	Alternate	Biallelic	Calling	Reference	Alternate	Reference	Alternate	Biallelic	Calling
Placenta 1	11	6	4	7	7	Het	17	30	12	62	0	Het
Placenta 2	5	0	12	6	3	Het	49	0	19	1	0	Het
Placenta 3	0	0	15	9	1	Het	1	25	6	67	0	Alt
Placenta 4	9	5	6	3	1	Het	6	30	18	27	0	Het

B

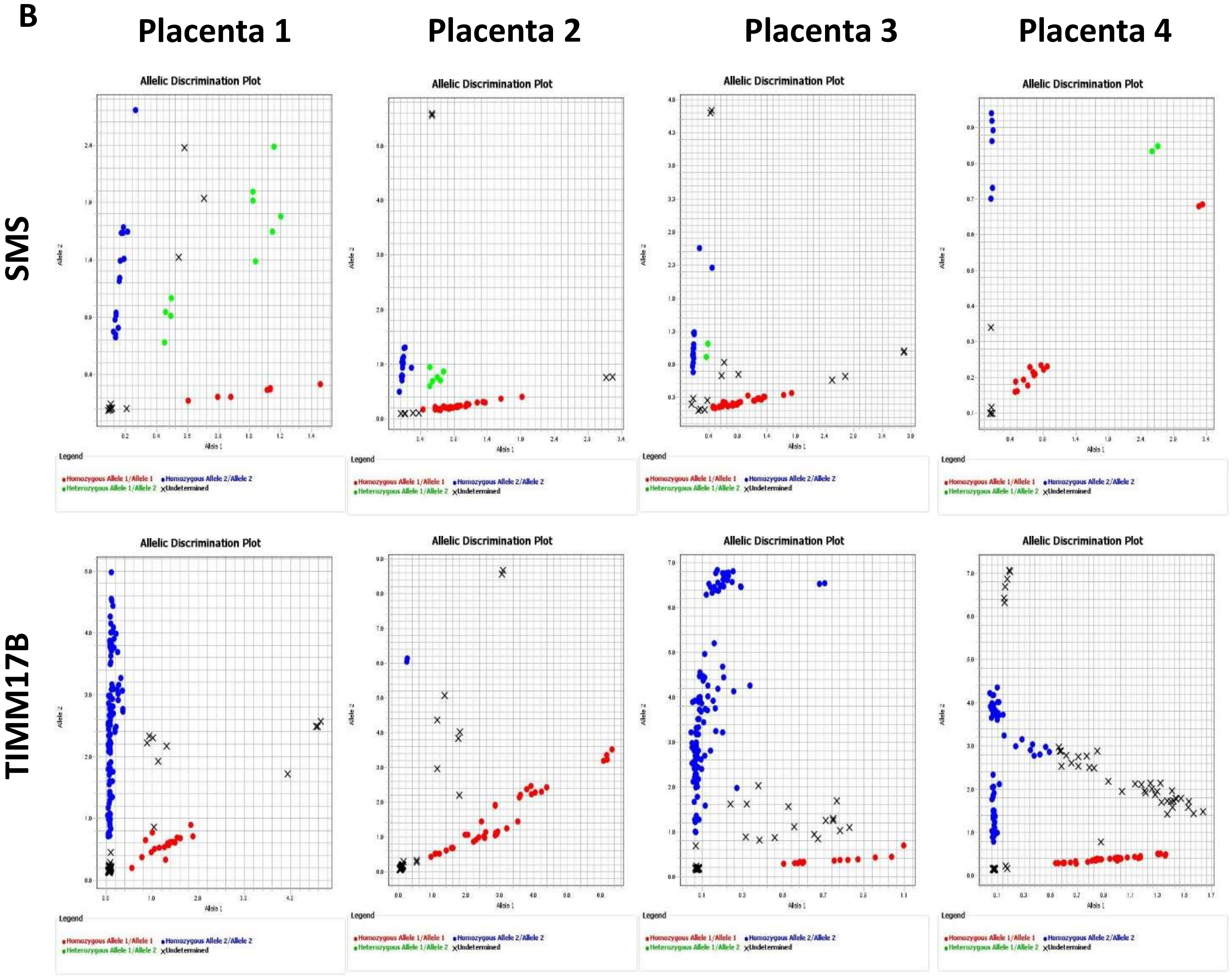

Supplemental Figure 1: Allelic discrimination of SMS and TIMM17B in isolated single nuclei. (A) Allelic discrimination plots demonstrating reference, alternative or biallelic expression of SMS and TIMM17B in single nuclei. (**B**) Summary table of SNPs called in each placenta.

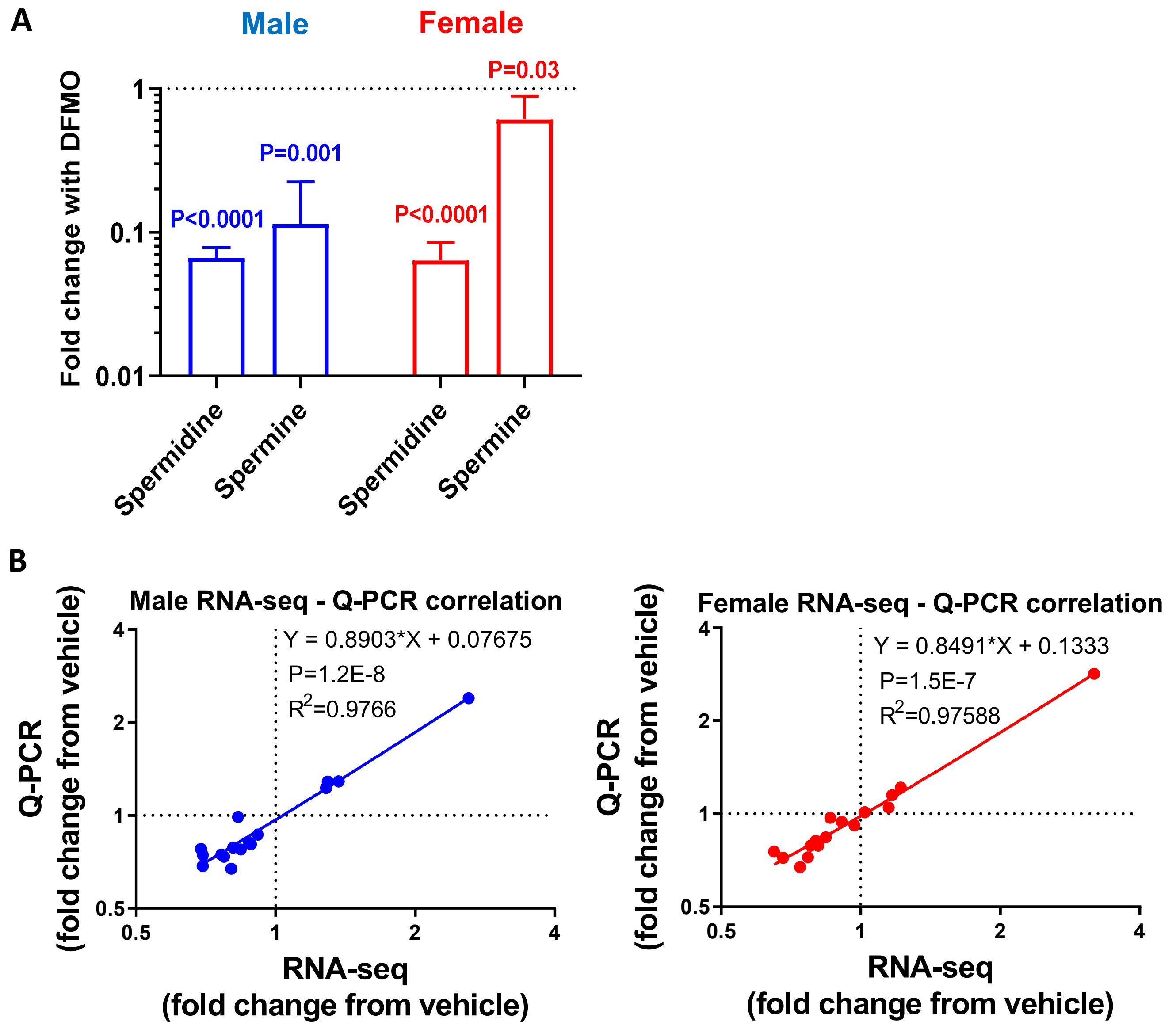

Supplemental Figure 2: Sex-specific effects of DFMO on intracellular polyamine levels and RNA-seq validation

(**A**) Effect of DFMO on polyamine levels in PHTs. (**B**) Validation of RNA-seq by Q-PCR in independent biological replicates. Each data point represents a separate gene (mean fold change), N=8-10 male PHTs and N=8-10 female PHTs.

A

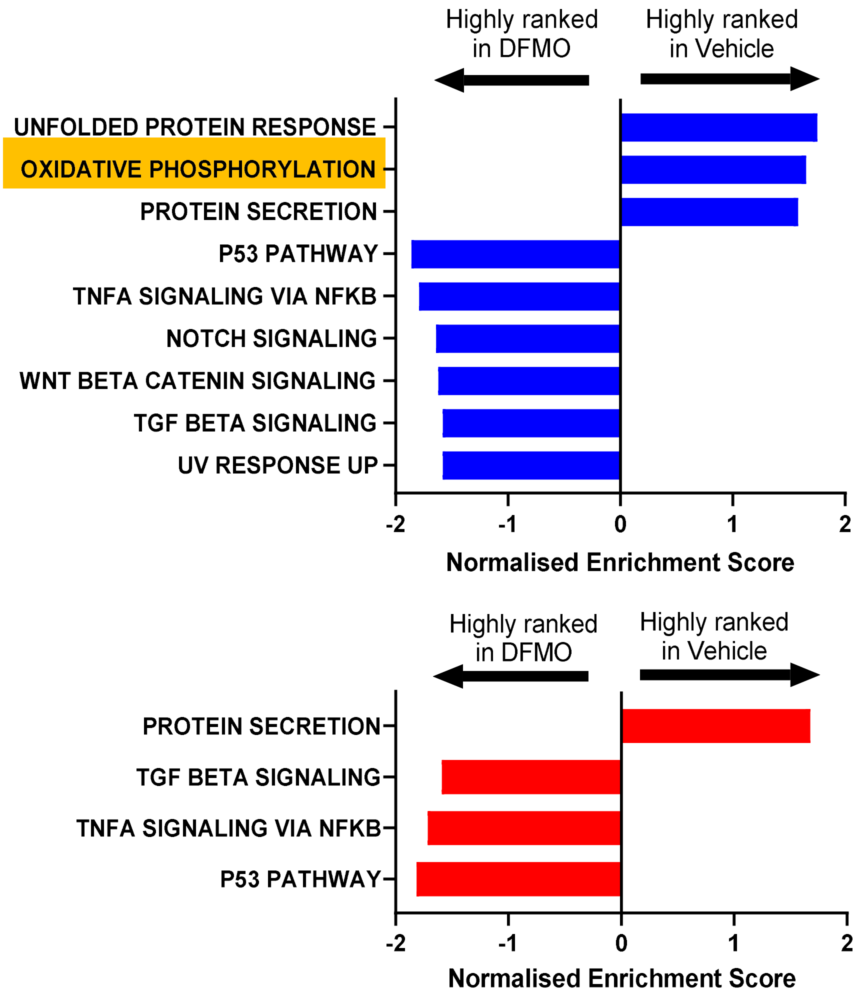

B

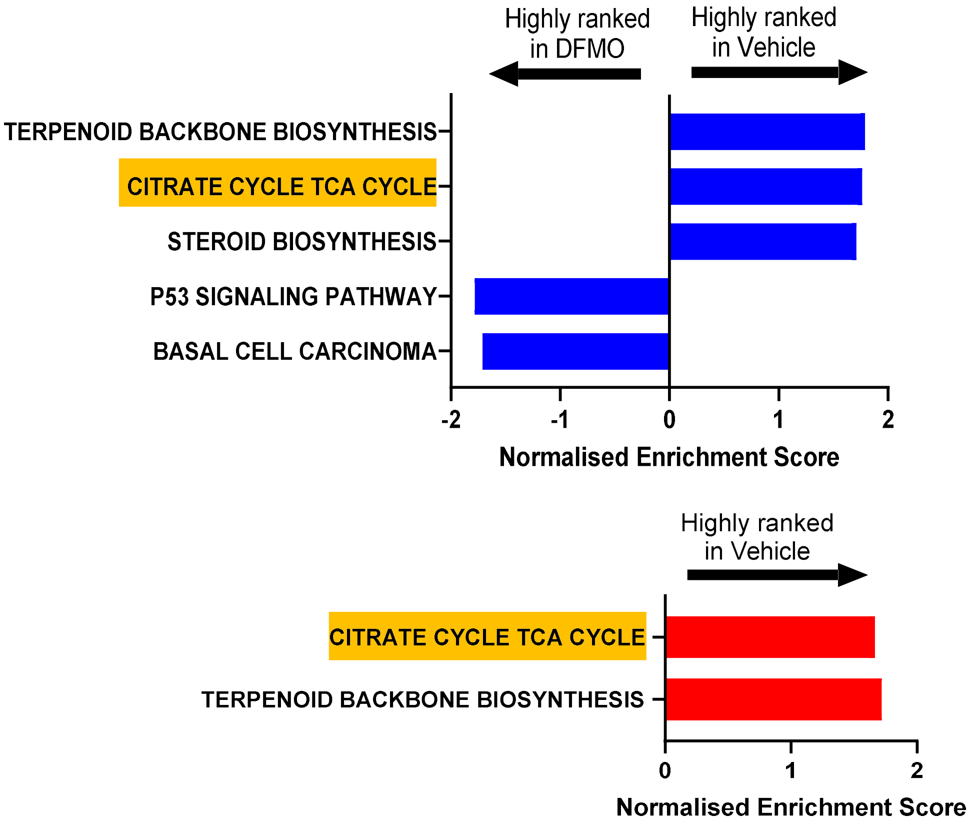

Supplemental Figure 3: Gene set enrichment analysis of PHTs following polyamine depletion
Number of GSEA classes in which DEGs are overrepresented by DFMO treatment (FDR-adjusted *P*-value <0.05) in Hallmarks and KEGG pathways. (**A**) Normalised Enrichment Scores of HALLMARK pathways. (**B**) Normalised Enrichment Scores of KEGG pathways. DEGs highly ranked in DFMO indicate upregulation with DFMO and DEGs highly ranked in vehicle indicate downregulation with DFMO. Blue indicates pathways enriched in male PHTs and red indicates pathways enriched in female PHTs.

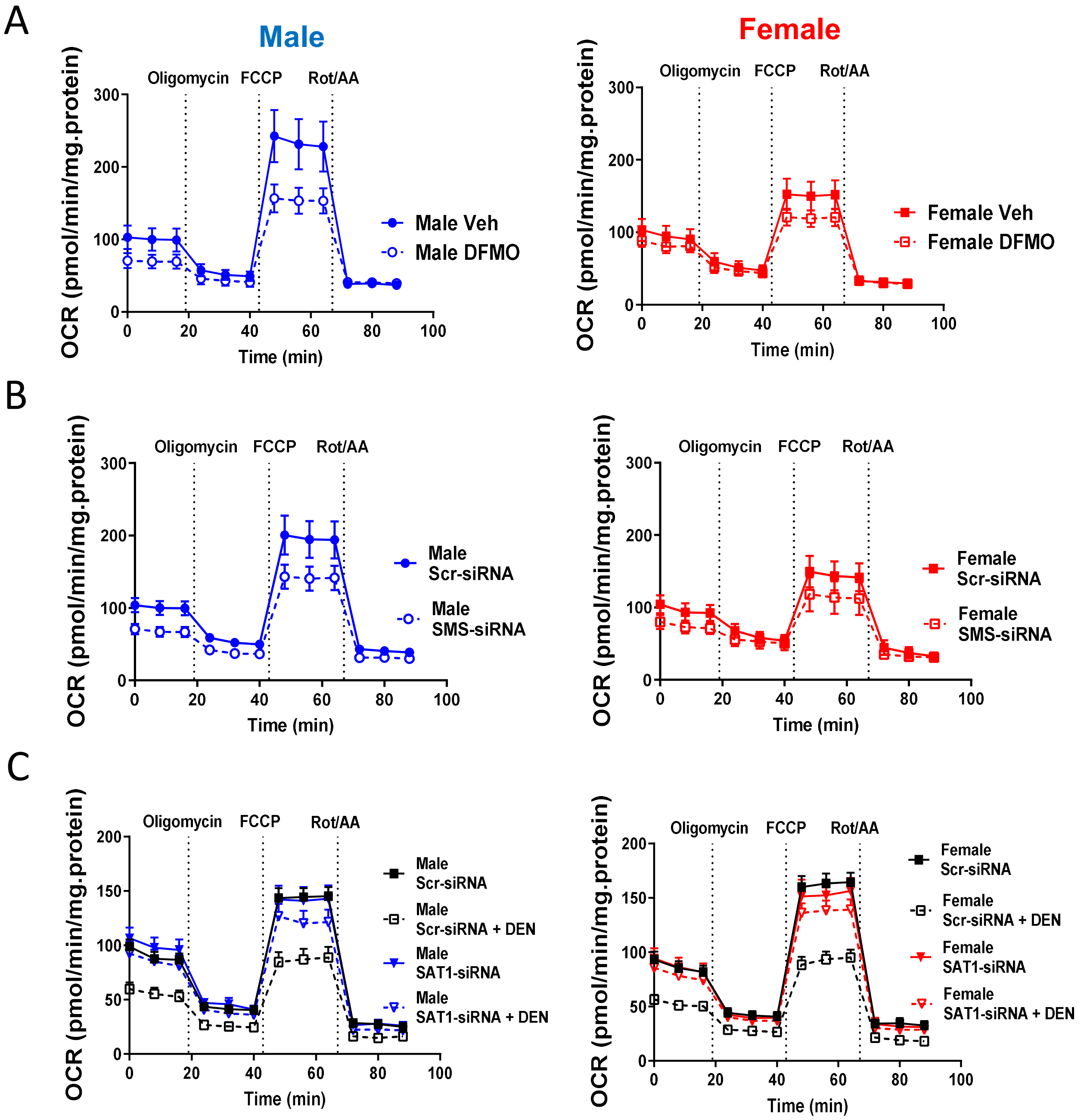

Supplemental Figure 4. Oxygen consumption rate following targeting of polyamine metabolism.

Oxygen consumption rate (OCR) in male and female PHTs following (**A**) DFMO treatment, (**B**) SMS silencing, (**C**) SSAT activation by DENSPM and reversal by SAT1 silencing. Mitochondrial respiration modulators (oligomycin, FCCP, Rotenone/Antimycin A [Rot/AA]) were injected at the indicated time points shown by the dotted lines. Graphs show mean±SEM, N=10 male PHTs, N=10 female PHTs. ND, not detected.

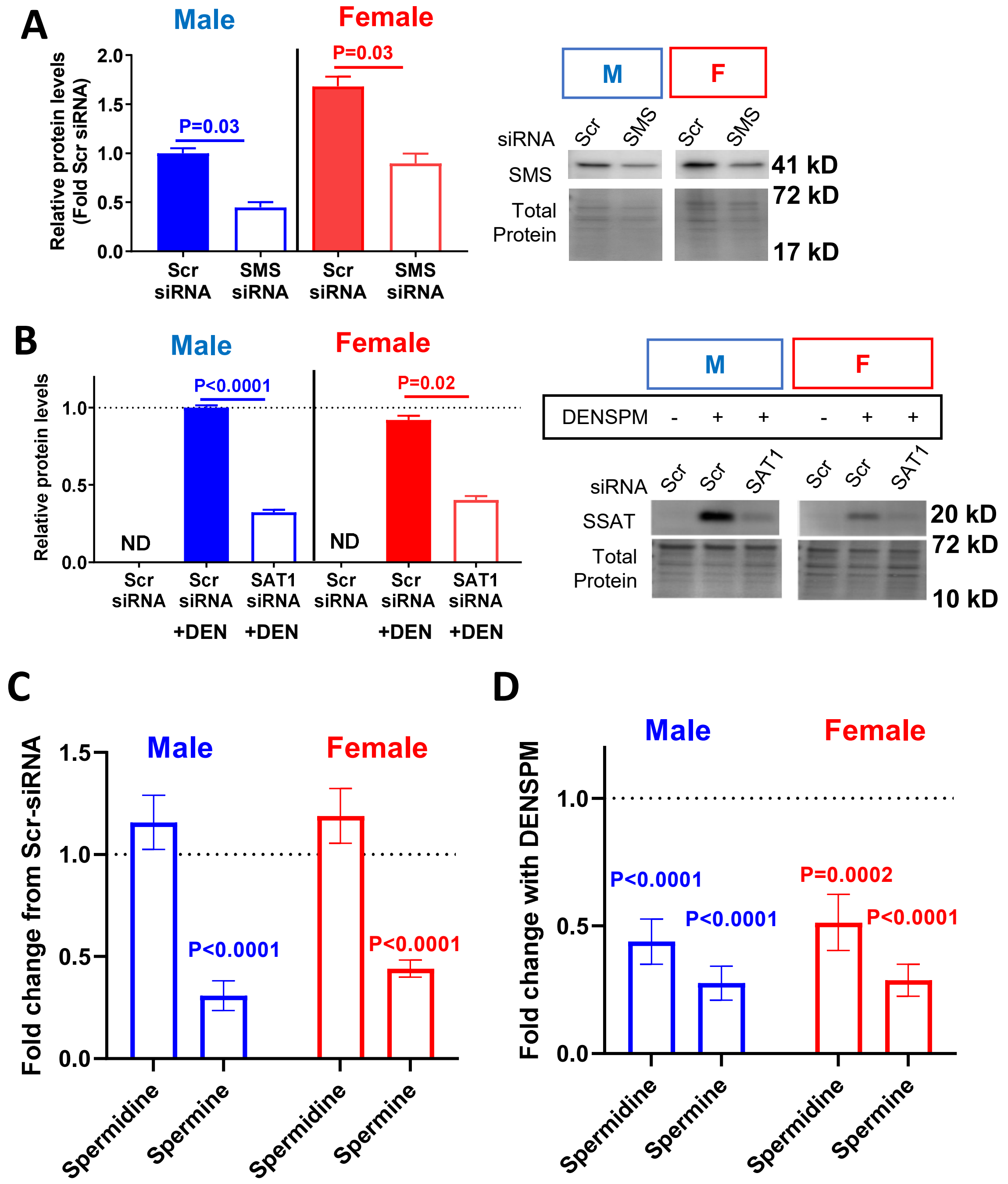

Supplemental Figure 5. Effects of targeting SMS and SSAT on polyamine levels
(**A**) SMS protein levels following siRNA silencing. (**B**) SSAT protein levels following DENSPM treatment with/without siRNA-silencing. Polyamine levels following (**C**) siRNA-mediated SMS silencing and (**D**) SSAT-induction with DENSPM.

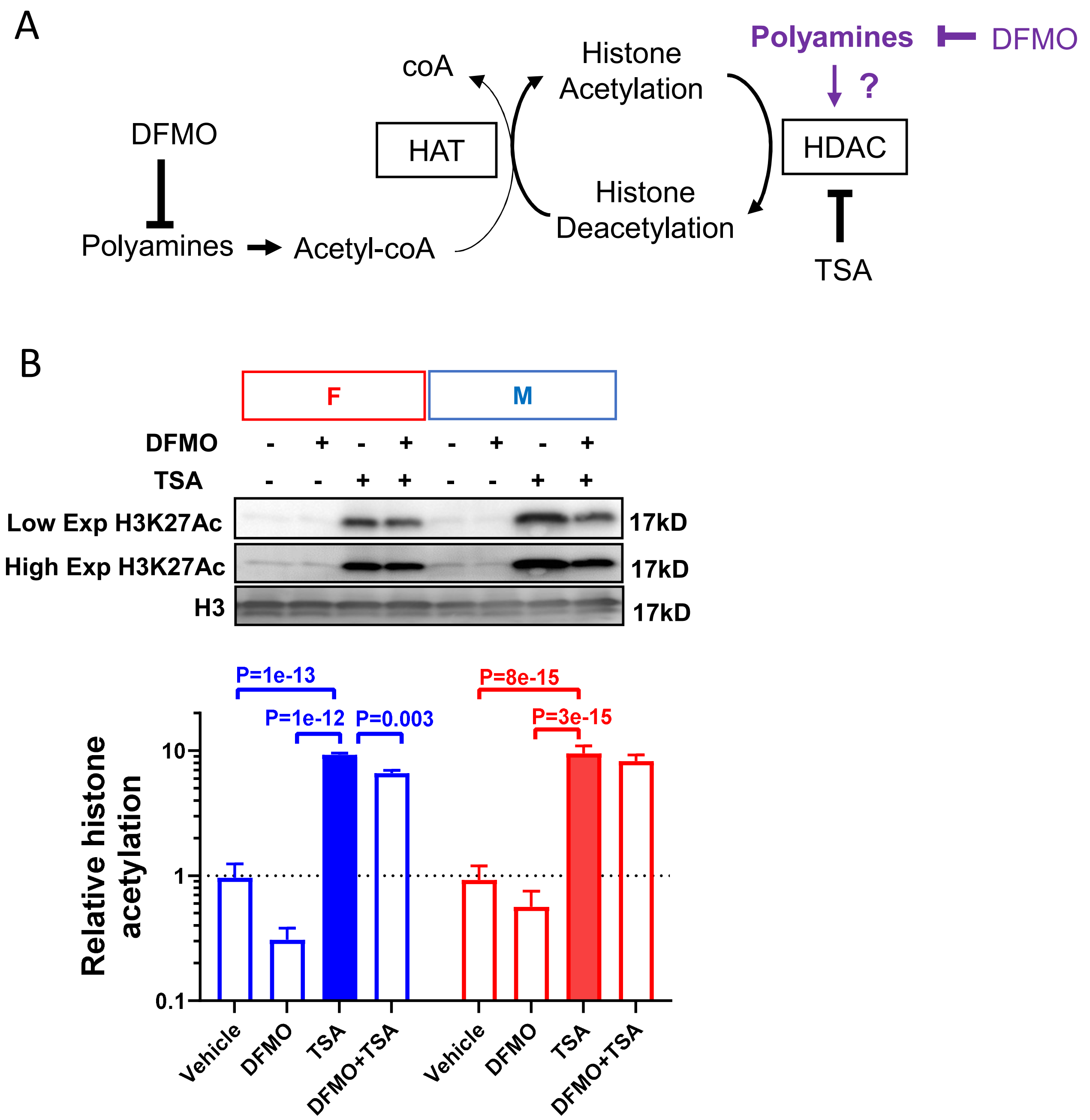

Supplemental Figure 6: Histone deacetylase activity is not involved in polyamine's effects on histone acetylation. (**A**) Model of the effect of polyamines on histone acetylation (shown in black) and alternative hypothesis (shown in purple). (**B**) Decrease in histone acetylation by DFMO is independent of HDAC inhibition by TSA. Blue bars indicate male PHTs and red bars indicate female PHTs. Bar graphs show mean±SEM, N=10 male PHTs and N=9 female PHTs.

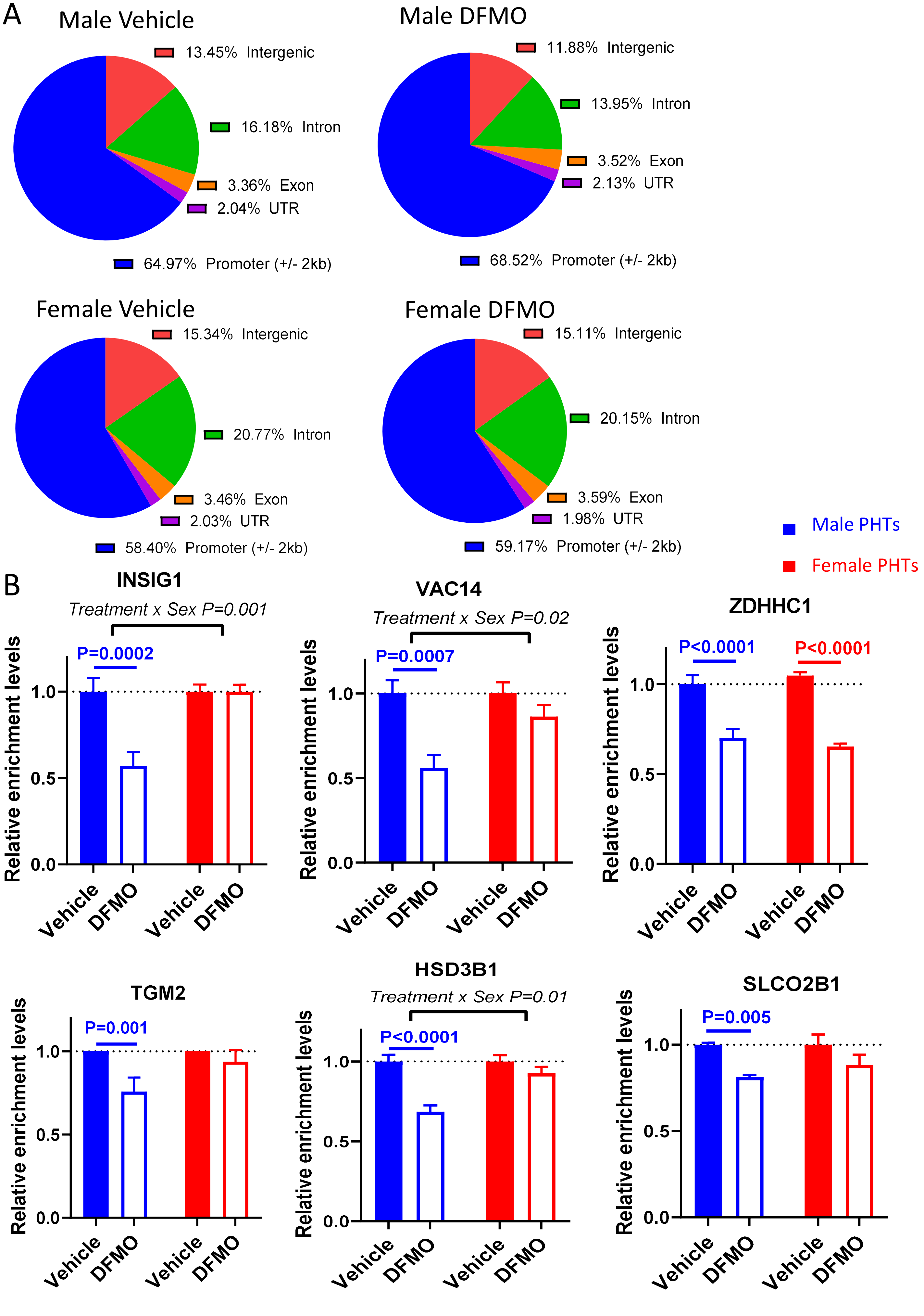

Supplemental Figure 7. ChIP-seq and validation by ChIP-QPCR

(**A**) H3K27Ac occupancy in genomic regions of PHT. (**B**) Validation of ChIP-seq results by ChIP-qPCR in independent biological replicates. Blue bars indicate male PHTs and red bars indicate female PHTs. Bar graphs show mean±SEM, N=5 male PHTs and N=5 female PHTs.

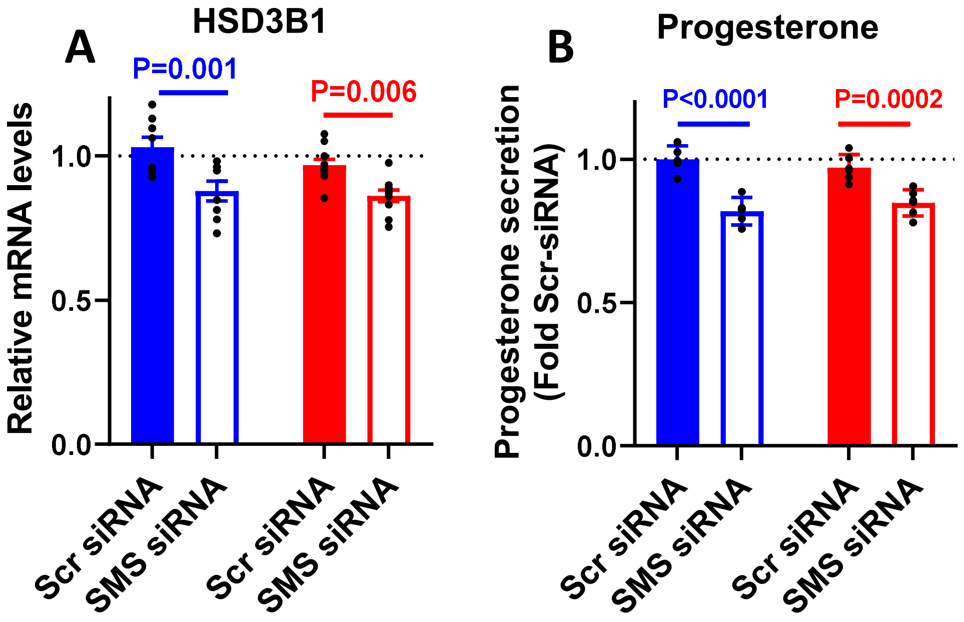

Supplemental Figure 8. SMS-silencing decreases HSD3B1 and progesterone release. (A) HSD3B1 mRNA and (B) progesterone secretion. Blue bars indicate male PHTs and red bars indicate female PHTs. Bar graphs show mean±SEM, N=5-8 male PHTs and N=5-10 female PHTs.
