# Supplementary material for: Placental sex-dependent spermine synthesis regulates trophoblast gene expression through acetyl-coA metabolism and histone acetylation
Source: Commun Biol. 2022 Jun 15;5:586. doi: 10.1038/s42003-022-03530-6 (PMC9200719; doi:10.1038/s42003-022-03530-6)
Supplement: Supplementary file 3 — Description of Additional Supplementary Files [file 42003_2022_3530_MOESM3_ESM.pdf]

Description of Additional Supplementary Files

File name: Supplementary Data 1

Description: Differentially expressed genes (DEGs) in male vehicle vs DFMO

File Name: Supplementary Data 2

Description: Differentially expressed genes (DEGs) in female vehicle vs DFMO

File Name: Supplementary Data 3

Description: Differentially binding regions (DBRs) in male vehicle vs DFMO

File Name: Supplementary Data 4

Description: Differentially binding regions (DBRs) in male vehicle vs DFMO

File Name: Supplementary Data 5

Description: List of primers

File Name: Supplementary Data 6

Description: Key Resources Table

File name: Supplementary Data 7

Description: Source Data

File name: Supplementary Data 8

Description: Uncropped western blots
