# Supplementary material for: Placental sex-dependent spermine synthesis regulates trophoblast gene expression through acetyl-coA metabolism and histone acetylation
Source: Commun Biol. 2022 Jun 15;5:586. doi: 10.1038/s42003-022-03530-6 (PMC9200719; doi:10.1038/s42003-022-03530-6)
Supplement: Supplementary file 11 — Supplemental Data 8 [file 42003_2022_3530_MOESM11_ESM.pdf]

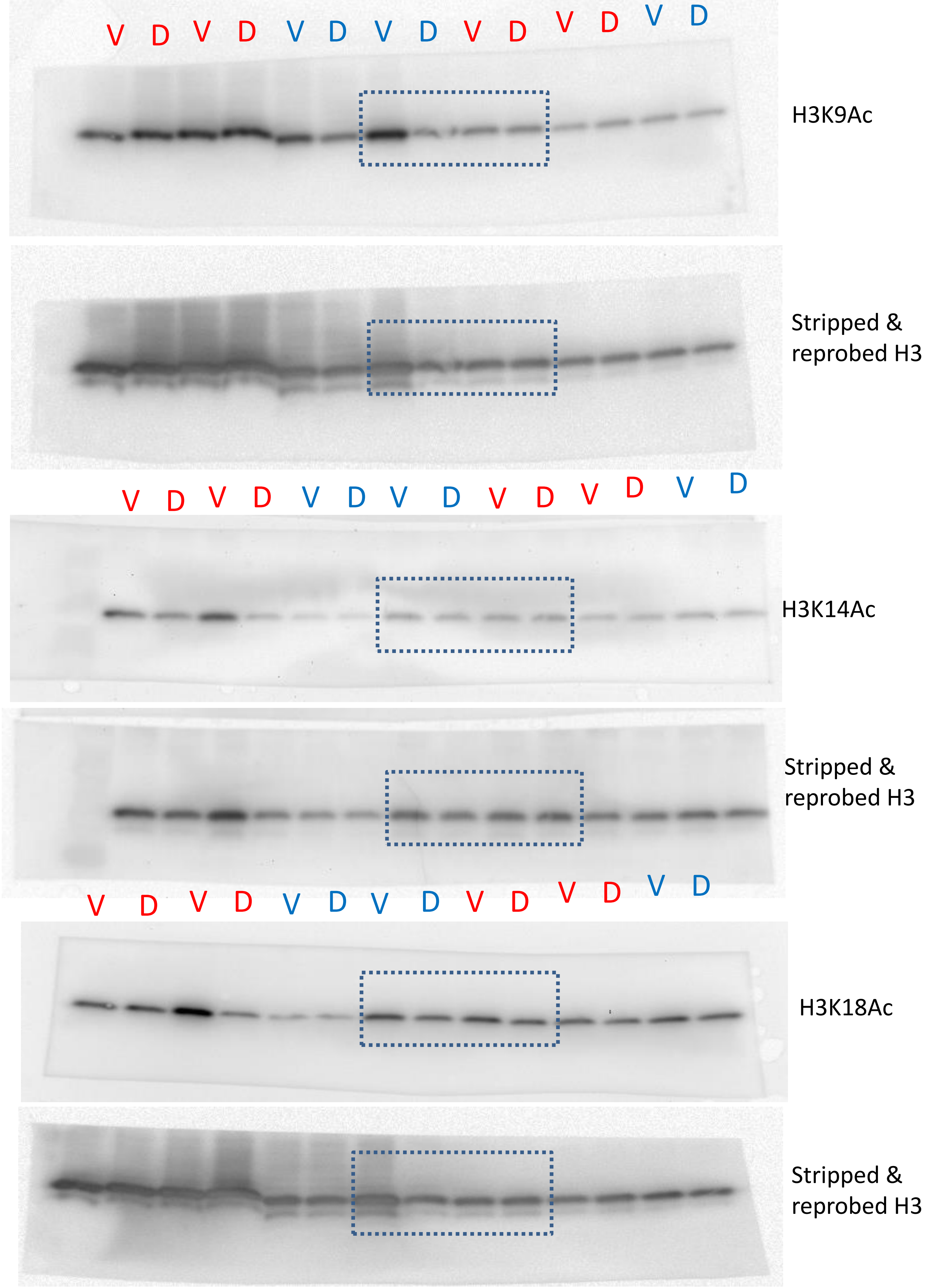

V D V D V D V D V D V D V D
H3K9Ac
Stripped & reprobed H3
V D V D V D V D V D V D V D
H3K14Ac
Stripped & reprobed H3
V D V D V D V D V D V D V D
H3K18Ac
Stripped & reprobed H3

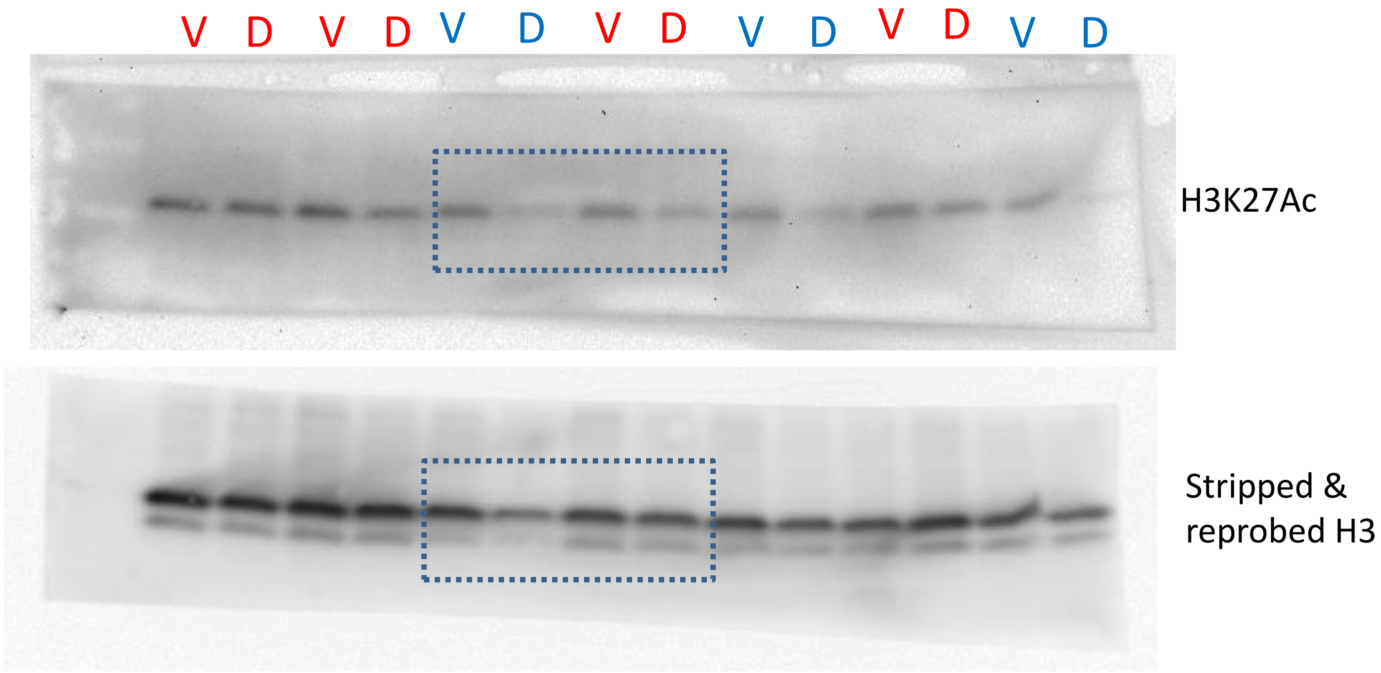

Uncropped blots from Figure 5B. V, vehicle; D, DFMO; blue, male trophoblasts; red, female trophoblasts

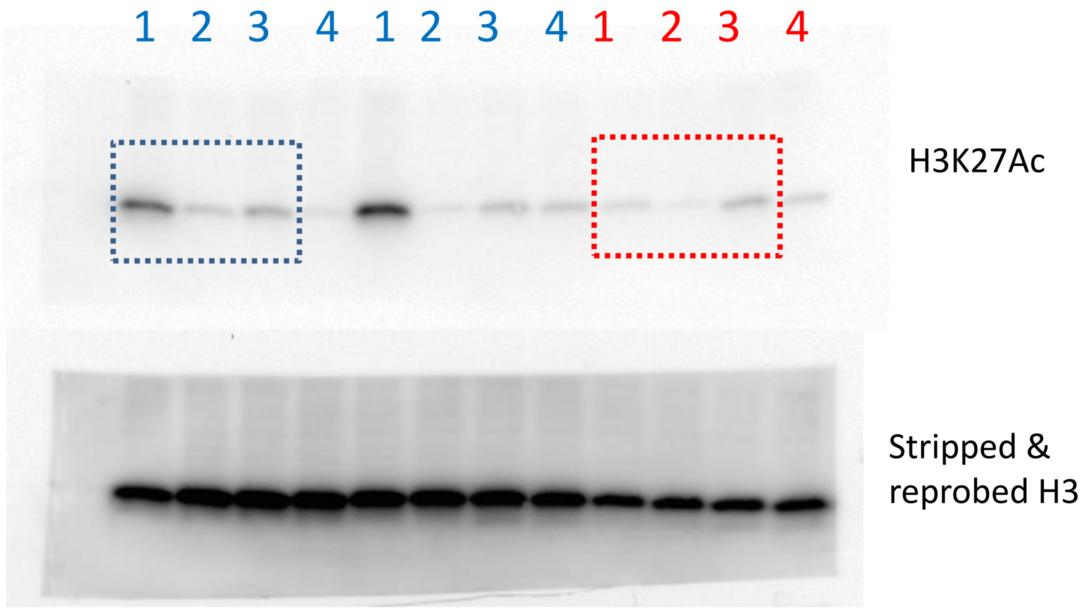

1: Scr; 2: Scr + DENSPM; 3: siSAT1+DENSPM; 4: siSAT1

Uncropped blots from Figure 5C. Blue numbers = male trophoblasts; red numbers = female trophoblasts

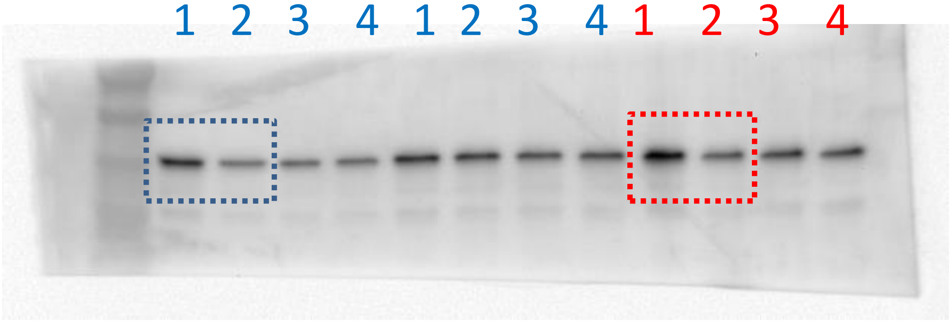

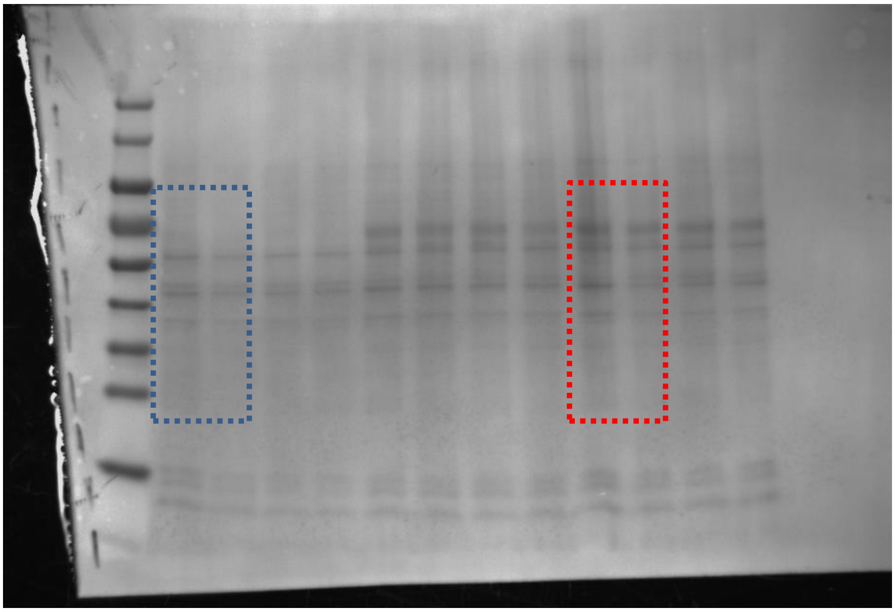

1: Scr; 2: siSMS#1; 3: siSMS#2 4: siSMS#3

Uncropped blots from Supplemental Figure 5A. Blue numbers = male trophoblasts; red numbers = female trophoblasts

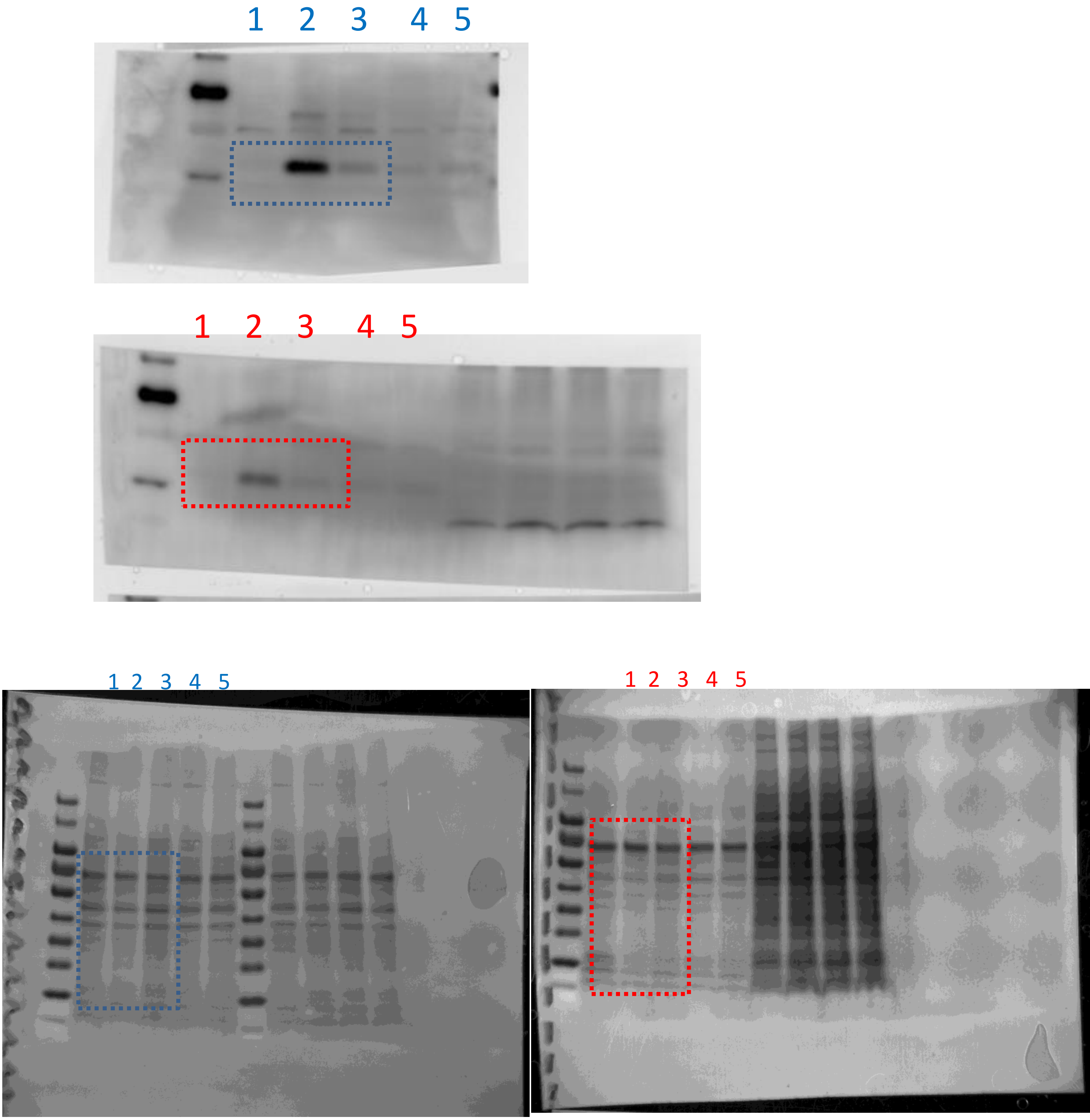

1: Scr; 2: Scr+DENSPM; 3: siSAT1#1 + DENSPM; 4: siSAT1#2 + DENSPM; 5: siSAT1 #3 + DENSPM

Uncropped blots from Supplemental Figure 5B. Blue numbers = male trophoblasts; red numbers = female trophoblasts

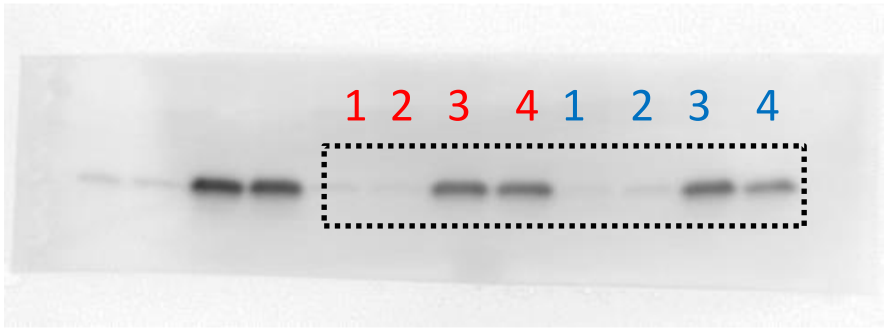

H3K27Ac low exposure

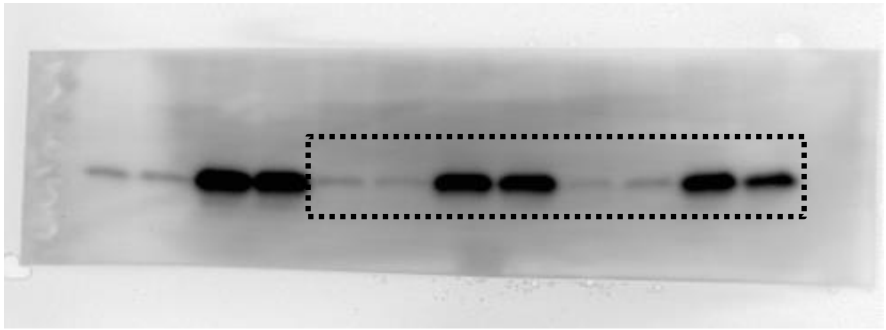

H3K27Ac high exposure

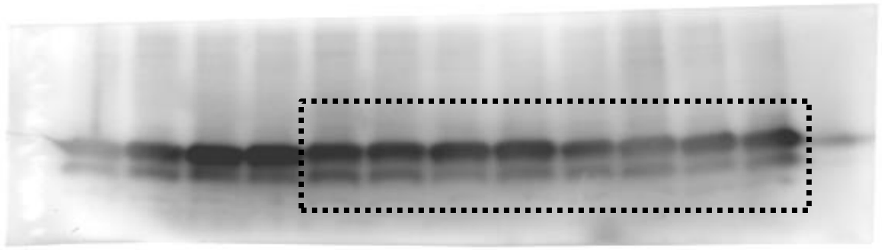

Reprobed H3

1: Veh, 2: DFMO, 3: TSA, 4: DFMO + TSA

Uncropped blots from Supplemental Figure 6B. Blue numbers = male trophoblasts; red numbers = female trophoblasts
