# Supplementary material for: Placental sex-dependent spermine synthesis regulates trophoblast gene expression through acetyl-coA metabolism and histone acetylation
Source: Commun Biol. 2022 Jun 15;5:586. doi: 10.1038/s42003-022-03530-6 (PMC9200719; doi:10.1038/s42003-022-03530-6)
Supplement: Supplementary file 12 — Reporting Summary [file 42003_2022_3530_MOESM12_ESM.pdf]

nature research

Corresponding author(s): COMMSBIO-21-3079A Irving Aye

Last updated by author(s): Apr 8, 2022

Reporting Summary

Nature Research wishes to improve the reproducibility of the work that we publish. This form provides structure for consistency and transparency in reporting. For further information on Nature Research policies, see our Editorial Policies and the Editorial Policy Checklist.

Statistics

For all statistical analyses, confirm that the following items are present in the figure legend, table legend, main text, or Methods section.

n/a	Confirmed	
☐	☒	The exact sample size (*n*) for each experimental group/condition, given as a discrete number and unit of measurement
☐	☒	A statement on whether measurements were taken from distinct samples or whether the same sample was measured repeatedly
☐	☒	The statistical test(s) used AND whether they are one- or two-sided<br>*Only common tests should be described solely by name; describe more complex techniques in the Methods section.*
☒	☐	A description of all covariates tested
☐	☒	A description of any assumptions or corrections, such as tests of normality and adjustment for multiple comparisons
☐	☒	A full description of the statistical parameters including central tendency (e.g. means) or other basic estimates (e.g. regression coefficient) AND variation (e.g. standard deviation) or associated estimates of uncertainty (e.g. confidence intervals)
☐	☒	For null hypothesis testing, the test statistic (e.g. *F*, *t*, *r*) with confidence intervals, effect sizes, degrees of freedom and *P* value noted<br>*Give P values as exact values whenever suitable.*
☒	☐	For Bayesian analysis, information on the choice of priors and Markov chain Monte Carlo settings
☒	☐	For hierarchical and complex designs, identification of the appropriate level for tests and full reporting of outcomes
☒	☐	Estimates of effect sizes (e.g. Cohen's *d*, Pearson's *r*), indicating how they were calculated

Our web collection on statistics for biologists contains articles on many of the points above.

Software and code

Policy information about availability of computer code

Data collection	Q-PCR and hetSNP allele typing analysis: QuantStudio Realtime PCR software (ThermoFisher Scientific)<br>Mass Spectrometry Spectra: ThermoExcalibur (ThermoFisher Scientific)<br>Metabolic phenotyping: Seahorse Wave Desktop v2.6.3.5 (Agilent)<br>Western Blot: Discovery Series Quantity One 1-D Analysis Software Version 4.6.8 (BioRad)<br>Microsoft Excel 2013 (Microsoft)
Data analysis	BBioinformatics software used:<br>GATK v3.4<br>FastQC (v0.11.4)<br>Salmon (v0.9.1)<br>DESeq2 (v1.18.1)<br>GSEA v.4.1.0<br>cutadapt (v2.7 with Python 3.6.8)<br>bowtie2 (v2.4.2)<br>sambamba (v0.7.1)<br>MACS2 (v.2.2.7.1)<br>ChIPseeker (v1.26)<br>DiffBind (v3.0.13)<br>R (v.4.0.3)

Bioconductor (v3.12)
Graph generation and statistical analyses: GraphPad Prism 9.2
Western blot data analysis: ImageJ 1.8.0
hetSNP allele typing analysis: Taqman Genotyper v.1.4

For manuscripts utilizing custom algorithms or software that are central to the research but not yet described in published literature, software must be made available to editors and reviewers. We strongly encourage code deposition in a community repository (e.g. GitHub). See the Nature Research guidelines for submitting code & software for further information.

Data

Policy information about availability of data

All manuscripts must include a data availability statement. This statement should provide the following information, where applicable:
- Accession codes, unique identifiers, or web links for publicly available datasets
- A list of figures that have associated raw data
- A description of any restrictions on data availability

RNA-sequencing and ChIP-sequencing data sets have been deposited in EBI ENA www.ebi.ac.uk/ena/data/view/PRJEB45391.
All source data for the figures are available in the Supplemental Information or Supplemental Data files. All other data (if any) are available upon reasonable request

Field-specific reporting

Please select the one below that is the best fit for your research. If you are not sure, read the appropriate sections before making your selection.

☒ Life sciences ☐ Behavioural & social sciences ☐ Ecological, evolutionary & environmental sciences

For a reference copy of the document with all sections, see nature.com/documents/nr-reporting-summary-flat.pdf

Life sciences study design

All studies must disclose on these points even when the disclosure is negative.

Sample size	We did not use statistical methods to calculate sample sizes, because the magnitudes of the effect sizes were not previously known. However, our sample sizes are similar to those reported in related publications in the field.
Data exclusions	No data was excluded
Replication	All experiments were replicated in 5-10 biological replicates.
Randomization	Randomization was not relevant to this study
Blinding	The investigators were not blinded to group allocation during experiments and outcome assessment due to the experimental design in which treatments and placental sex were purposefully selected for analysis. RNA-seq and ChIP-seq was performed blind to the experimental groups.

Reporting for specific materials, systems and methods

We require information from authors about some types of materials, experimental systems and methods used in many studies. Here, indicate whether each material, system or method listed is relevant to your study. If you are not sure if a list item applies to your research, read the appropriate section before selecting a response.

Materials & experimental systems

n/a	Involved in the study	
☐	☒	Antibodies
☒	☐	Eukaryotic cell lines
☒	☐	Palaeontology and archaeology
☒	☐	Animals and other organisms
☐	☒	Human research participants
☐	☒	Clinical data
☒	☐	Dual use research of concern

Methods

n/a	Involved in the study	
☐	☒	ChIP-seq
☒	☐	Flow cytometry
☒	☐	MRI-based neuroimaging

Antibodies

Antibodies used	Rabbit monoclonal anti-spermine synthase (clone EPR9252(B)) Abcam ab156879<br>Rabbit polyclonal anti-SAT1 Abcam ab105220<br>Rabbit monoclonal anti-histone H3 (clone D1H2) Cell Signaling Technology 4499

	Rabbit monoclonal anti-acetyl-histone H3 Lys9 (clone C5B11) Cell Signaling Technology 9649<br>Rabbit monoclonal anti-acetyl-histone H3 Lys14 (clone D4B9) Cell Signaling Technology 7627<br>Rabbit monoclonal anti-acetyl-histone H3 Lys18 (clone D8Z5H) Cell Signaling Technology 13998<br>Rabbit monoclonal anti-acetyl-histone H3 Lys27 (clone D5E4) Cell Signaling Technology 8173<br>HRP-conjugated anti-rabbit IgG Cell Signaling Technology 7074<br>Rabbit polyclonal anti-acetyl-histone H3 Lys27 (ChIP-grade) Abcam ab4729
Validation	anti-spermine synthase: PMID: 33586680, 31391464, 29997303, 26910893<br>anti-SAT1 RRID:AB_11127304<br>anti-histone H3 (clone D1H2): RRID:AB_10544537<br>anti-acetyl-histone H3 Lys9 (clone C5B11): RRID:AB_823528<br>anti-acetyl-histone H3 Lys14 (clone D4B9): RRID:AB_10839410<br>anti-acetyl-histone H3 Lys18 (clone D8Z5H): RRID:AB_2783723<br>anti-acetyl-histone H3 Lys27 (clone D5E4): RRID:AB_10949503<br>anti-rabbit IgG: RRID:AB_2099233<br>anti-acetyl-histone H3 Lys27 (ChIP-grade): RRID:AB_2118291

Human research participants

Policy information about studies involving human research participants

Population characteristics	The POP study population was an unselected cohort of nulliparous women with a singleton pregnancy who delivered in Cambridge, UK. The cohort has been described in detail in our previous papers (Sovio et al, Lancet 2015;386(10008):2089-2097; Gaccioli et al, Placenta 2017;59(Supp1):S17-S25; Gong et al, JCI Insight 2018;3(13):e120723).
Recruitment	Participants were recruited at their dating scan appointment at around 12 weeks of gestation. The representativeness of the recruited population has been described in our previous publication (Sovio et al, Lancet 2015;386(10008):2089-2097).
Ethics oversight	Ethical approval was obtained from the Cambridgeshire 2 Research Ethics Committee (REC ID 07/H0308/163)

Note that full information on the approval of the study protocol must also be provided in the manuscript.

Clinical data

Policy information about clinical studies

All manuscripts should comply with the ICMJE guidelines for publication of clinical research and a completed CONSORT checklist must be included with all submissions.

Clinical trial registration	NA
Study protocol	NA
Data collection	NA
Outcomes	NA

ChIP-seq

Data deposition

☒ Confirm that both raw and final processed data have been deposited in a public database such as GEO.

☒ Confirm that you have deposited or provided access to graph files (e.g. BED files) for the called peaks.

Data access links<br>*May remain private before publication.*	https://www.ebi.ac.uk/ena/data/view/PRJEB45391 (raw data)<br>https://sung.github.io/placenta-polyamine-2022 (processed data)
Files in database submission	ENA data includes reads (fastq), signals (bw) and peaks (bed) for all replicates, treatments and inputs.
Genome browser session<br>(e.g. UCSC)	*Provide a link to an anonymized genome browser session for "Initial submission" and "Revised version" documents only, to enable peer review. Write "no longer applicable" for "Final submission" documents.*

Methodology

Replicates	Biological replicates of male and female placentas, N=6 male placental trophoblasts, N=5 female placental trophoblasts comparing vehicle vs DFMO treatment. Input DNA from matching placental trophoblasts was used as controls
Sequencing depth	Single end 50bp. Total number of reads: 379108610, Uniquely mapped reads: 275158075

Antibodies	Rabbit polyclonal anti-acetyl-histone H3 Lys27 (ChIP-grade) Abcam ab4729
Peak calling parameters	Sequencing reads were mapped to the human genome reference GRCh38 version using bowtie2 (v2.4.2) with the default parameters followed by removing duplicate reads using sambama (v.0.7.1). For peak callings, we used MACS2 (v.2.2.7.1) with the following parameters: --treatment $TREATMENT-SAMPLE.bam --control $MERGED_CONTROL_INPUT.bam --nomodel --extsize 148 --bdg. We merged BAM files of the inputs (i.e. controls) that match with the same sex-treatment types of the ChIP-Seq files.
Data quality	We used only high quality reads by removing primer sequences and poor quality bases from the sequencing reads. We also removed duplicate reads using sambamba (v.0.7.1). We detected 71231 consensus peaks with a FDR 5%.
Software	Primer sequences and poor quality bases were trimmed from the sequencing reads using cutadapt (v2.7 with Python 3.6.8)<br>Mapping to GRCh38 version human genome using bowtie2 (v2.4.2)<br>Duplicated reads were discarded using sambamba (v0.7.1)<br>Peaks called using MACS2 (v.2.2.7.1)<br>Peaks annotated using ChIPseeker (v1.26)<br>Differentially bound regions (vehicle vs DFMO) DiffBind (v3.0.13) using background normalization
